# MEF2A alters the proliferation, inflammation-related gene expression profiles and its silencing induces cellular senescence in human coronary endothelial cells

**DOI:** 10.1186/s12867-019-0125-z

**Published:** 2019-03-18

**Authors:** Yujuan Xiong, Lin Wang, Wenyi Jiang, Lihua Pang, Weihua Liu, Aiqun Li, Yun Zhong, Wenchao Ou, Benrong Liu, Shi-ming Liu

**Affiliations:** 1 Guangzhou Institute of Cardiovascular Disease, Guangdong Key Laboratory of Vascular Diseases, State Key Laboratory of Respiratory Disease, The Second Affiliated Hospital, Guangzhou Medical University, No. 250 Changgang Dong Road, Guangzhou, 510260 Guangdong People’s Republic of China; 2 Department of Laboratory Medicine, The Second Affiliated Hospital of Guangzhou University of Chinese Medicine, 111 Dade Road, Guangzhou, 510120 People’s Republic of China

**Keywords:** Myocyte enhancer factor 2A, Human coronary artery endothelial cells, Vascular endothelial dysfunction, Senescence, Cardiovascular disease, PIK3CG

## Abstract

**Background:**

Myocyte enhancer factor 2A (MEF2A) plays an important role in cell proliferation, differentiation and survival. Functional deletion or mutation in MEF2A predisposes individuals to cardiovascular disease mainly caused by vascular endothelial dysfunction. However, the effect of the inhibition of MEF2A expression on human coronary artery endothelial cells (HCAECs) is unclear. In this study, expression of MEF2A was inhibited by specific small interference RNA (siRNA), and changes in mRNA profiles in response to MEF2A knockdown were analyzed using an Agilent human mRNA array.

**Results:**

Silencing of MEF2A in HCAECs accelerated cell senescence and suppressed cell proliferation. Microarray analysis identified 962 differentially expressed genes (DEGs) between the MEF2A knockdown group and the negative control group. Annotation clustering analysis showed that the DEGs were preferentially enriched in gene ontology (GO) terms and Kyoto Encyclopedia of Genes and Genomes (KEGG) pathways related to proliferation, development, survival, and inflammation. Furthermore, 61 of the 578 downregulated DEGs have at least one potential MEF2A binding site in the proximal promoter and were mostly enriched in the GO terms “reproduction” and “cardiovascular.” The protein–protein interaction network analyzed for the downregulated DEGs and the DEGs in the GO terms “cardiovascular” and “aging” revealed that PIK3CG, IL1B, IL8, and PRKCB were included in hot nodes, and the regulation of the longevity-associated gene PIK3CG by MEF2A has been verified at the protein level, suggesting that PIK3CG might play a key role in MEF2A knockdown induced HCAEC senescence.

**Conclusions:**

MEF2A knockdown accelerates HCAEC senescence, and the underlying molecular mechanism may be involved in down-regulation of the genes related with cell proliferation, development, inflammation and survival, in which PIK3CG may play a key role.

**Electronic supplementary material:**

The online version of this article (10.1186/s12867-019-0125-z) contains supplementary material, which is available to authorized users.

## Background

MEF2A is one of the members of the MEF2 family that belongs to the MADS-box superfamily and shares homology in a 58-amino acid domain that mediates DNA binding and dimerization [1]. An MEF2A-encoded protein can act as a homodimer or heterodimer and is involved in several cellular processes, including neuronal differentiation, muscle development, cell growth control, and apoptosis [2]. MEF2A has an essential DNA-binding site in the MyoDa gene control region and is a transcription factor that activates many muscle-specific, growth factor-induced, and stress-induced genes [3, 4]. The critical roles of MEF2A in myocardial development have been confirmed in many studies. Naya et al. observed that mice lacking Mef2a die suddenly within the first week of life and exhibit pronounced right ventricle dilation, myofibrillar fragmentation, mitochondrial disorganization, and fetal cardiac gene program activation [5]. Chen et al. found that MEF2A participates in the pathway mediating cardiomyocyte development by AKT2 [6]. The association of genetic variations in the coding sequence of MEF2A with coronary artery disease (CAD) has also been broadly investigated. The deletion of seven amino acids disrupts the nuclear localization of MEF2A and reduces the MEF2A-mediated activation of transcription, and this deletion is co-segregated with premature CAD in a family lineage [7]. Another study has reported that a novel 6 bp deletion in exon 11 of MEF2A is closely associated with premature CAD [8]. Nonsynonymous mutation or variation in the number of CAG or CCG repeats is correlated with susceptibility to CAD in some populations [9, 10]. However, the roles of MEF2A variants in CAD are controversial because not all studies can show the association between these variants and CAD [11–15]. The inflammation and dysfunction of vascular endothelial cells or smooth muscle cells are implicated in the pathogenesis of CAD. MEF2A promotes vascular smooth muscle cell (VSMC) senescence and regulates H_2_O_2_-induced VSMC senescence by interacting with miR-143, and MEF2A inhibition induces the phenotypic switching of VSMCs, leading to increased cell proliferation and migration [16, 17]. Kim et al. demonstrated that the activity of MEF2 (MEF2A and MEF2C) is remarkably impaired in pulmonary arterial endothelial cells derived from the subjects with pulmonary arterial hypertension (PAH), and the restoration of the impaired function of endothelial MEF2 rescues PAH [18]. Silencing of MEF2A in apoE^−/−^ mice by using lentiviral shRNA considerably reduces plaque collagen content and fibrous cap thickness and increases plaque area; conversely, silencing of MEF2A has no obvious effect on plaque lipid content [19]. These studies have suggested that MEF2A plays important roles in cardiomyocytes, VSMCs, and pulmonary arterial endothelial cells, but its functions in coronary artery endothelial cells are unclear. In this study, we found that the inhibition of MEF2A by siRNA promoted HCAEC senescence. To explore the transcriptional change in the downstream molecular network in response to MEF2A knockdown, we conducted mRNA microarray analysis and revealed that many specific pathways might be regulated by MEF2A in HCAECs. We also described a downstream molecular network controlled by MEF2A.

## Results

### Inhibition of MEF2A in HCAECs promoted cellular senescence and suppressed proliferation

**Fig. 1:**
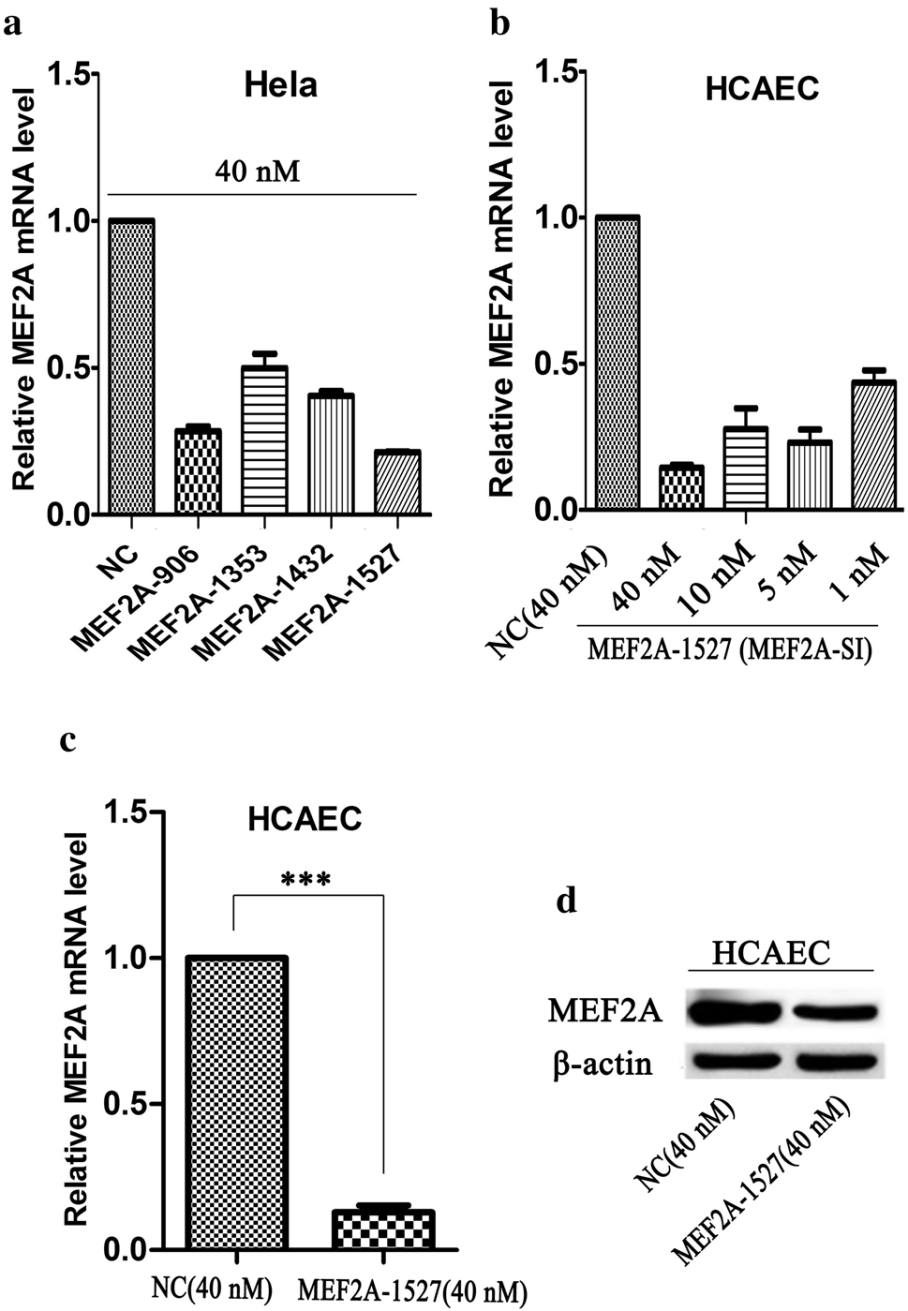
MEF2A was inhibited by siRNA on mRNA and protein level. **a** The inhibition efficiency of four pairs of MEF2A specific siRNA. **b** The inhibition efficiency of different concentration of MEF2A-1527. **c**, **d** Variation of the expression of MEF2A at mRNA and protein levels in HCAEC transfected with MEF2A-1527 at a final concentration of 40 nM

**Fig. 2:**
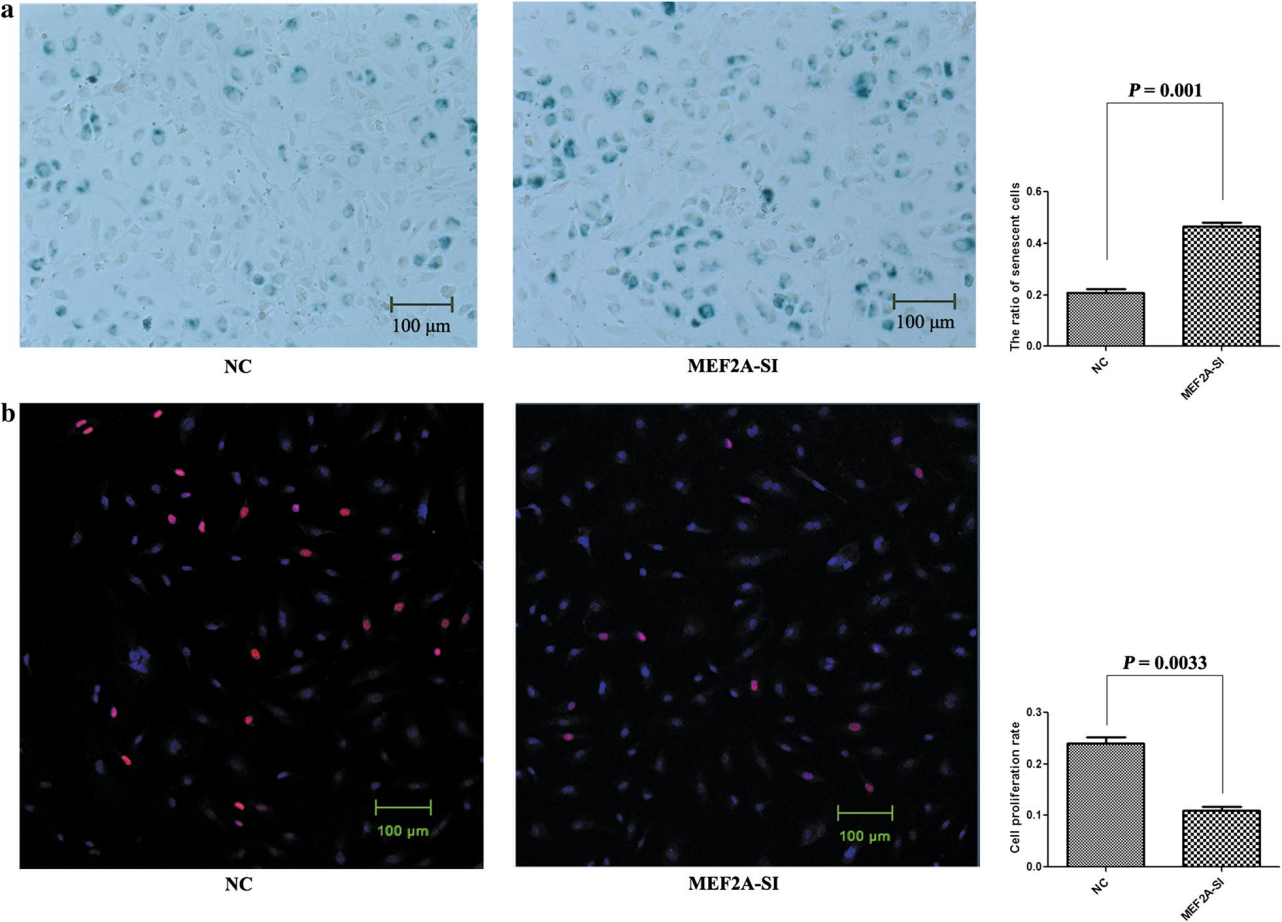
Alteration in phenotype of HCAEC in response to MEF2A knockdown. NC: negative control; MEF2A-SI: MEF2A silencing. **a** Senescent cells were detected using SA-β-Gal staining at 96 h after transfection and senescent cells were stained blue. **b** The proliferative capability of cells were tested by using EdU staining at 96 h after transfection and the cells stained purple have proliferative activity

In this study, we attempted to understand the effect of inhibiting the MEF2A expression on HCAECs. Four pairs of MEF2A-specific siRNA were designed to suppress the MEF2A expression, and MEF2A-1527 showed the highest inhibitory activity on the expression of MEF2A (Fig. 1a). MEF2A-1527 had a significant inhibitory effect on MEF2A when it was transfected at different concentrations in HCAEC (Fig. 1b), and a final concentration of 40 nM MEF2A-1527 remarkably inhibited MEF2A at both mRNA (Fig. 1c) and protein (Fig. 1d) levels in HCAEC. The results of senescence-associated β-galactosidase (SA-β-Gal) staining and EdU staining showed that MEF2A knockdown accelerates HCAEC senescence (Fig. 2a) and inhibites cell proliferation (Fig. 2b).

### Identification of DEGs in response to MEF2A knockdown in HCAECs

**Fig. 3:**
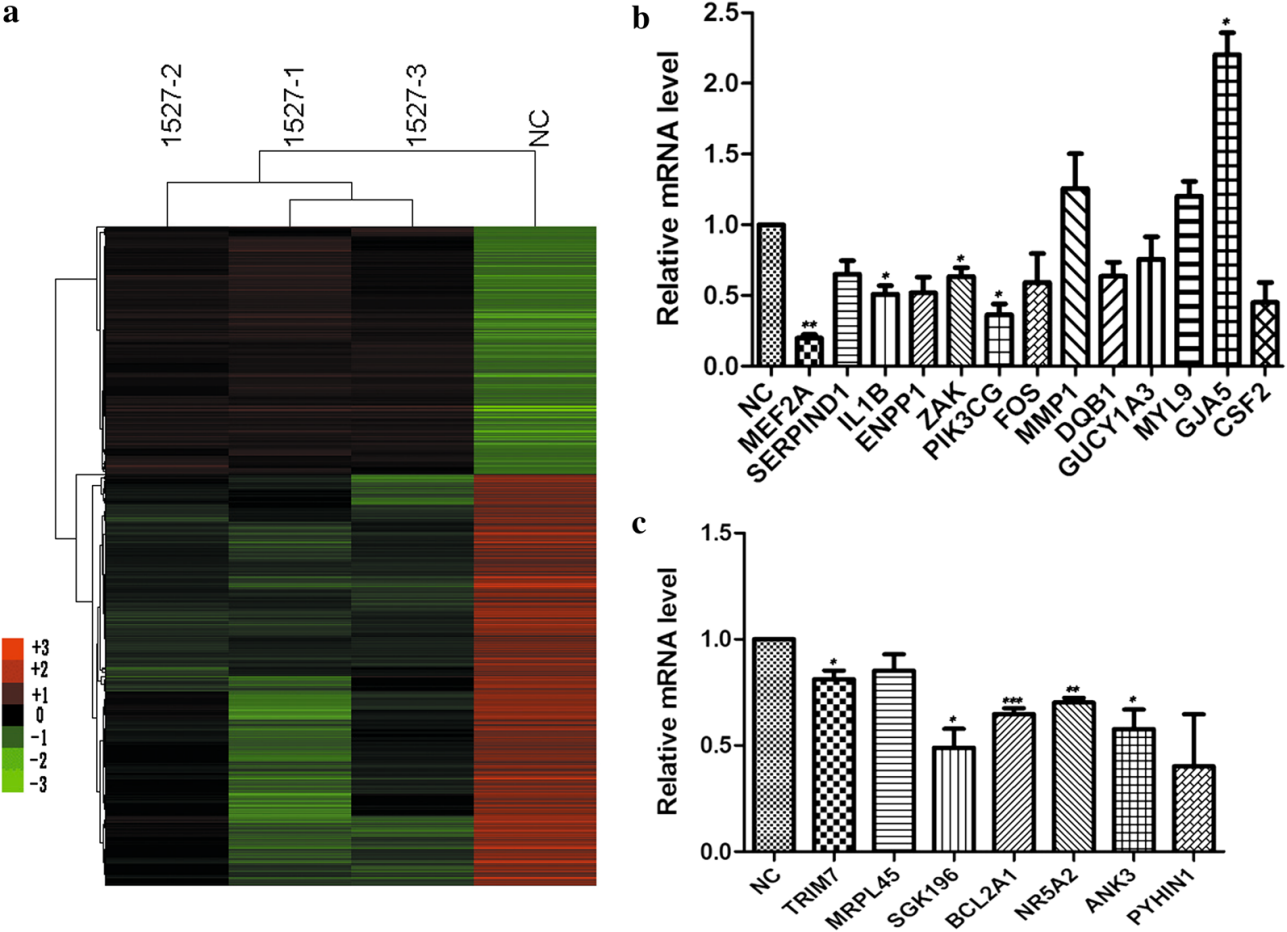
DEGs between MEF2A silencing group and NC-group. **a** The heatmap of the DEGs. Three groups of HCAEC transfected with MEF2A-1527 were respectively indicated with 1527-1, 1527-2 and 1527-3. The mixed sample with three groups of HCAEC transfected with negative control siRNA was defined as NC. **b**, **c** The selected DEGs were validated with real-time quantitative PCR. The relative expression of each gene was calculated according to 2^−((Cte1−Cte0) −(Ctn1−Ctn0))^, here, C_te1_ and C_te0_ respectively indicate the C_t_ value of the target gene and the internal reference gene (beta-actin) in MEF2A siRNA-groups; C_tn1_ and C_tn0_ respectively indicate the C_t_ value of the target gene and beta-actin in the NC groups. The relative expression of all selected genes was normalized to “1” in the NC groups. **c** These genes have at least one potential MEF2A binding site in its proximal promoter. *p < 0.05, **p < 0.01, ***p < 0.001 are significant

The MEF2A-specific siRNA group and the negative control group were subjected to genome-wide expression microarray analyses to determine the underlying regulatory network in response to the inhibition of MEF2A. The raw microarray data obtained in this experiment were deposited in Gene Expression Omnibus (GEO) [20] with an accession number of GSE114114. A total of 1363 genes had more than twofold changes in the expression between the MEF2A knockdown group and the NC group, and the difference in the gene expression patterns could be seen in the heat map (Fig. 3a). The DEGs, namely, MEF2A, SERPINP, IL1B, ENPP1, ZAK, PIK3CG, FOS, MMP1, HLA-DQB1, GUCY1A3, MYL9, GJA5, and CSF2, were selected for real-time q-PCR validation. Up-regulation of MMP1 and MYL9 and downregulation of the other genes except GJA5 in the MEF2A-siRNA group were observed in qPCR and microarray. The microarray result showed that GJA5 was significantly down-regulated, but the qPCR result revealed that GJA5 was up-regulated in the MEF2A-siRNA group (Fig. 3b).

Of the down-regulated DEGs, 61 were predicted with at least one MEF2A binding site in the proximal promoter (500 bp region upstream from the transcription start site) (Additional file 1: Table S1). Database for Annotation, Visualization, and Integrated Discovery (DAVID) analysis of the 61 genes indicated that many genes were significantly enriched in the terms “reproduction” and “cardiovascular” in the GAD_DISEASE_CLASS category. The terms “secreted,” “disulfide bond,” “signal peptide,” and “zinc finger region:B box-type” were the most significantly enriched (Table 1). A few of these DEGs were selected for qPCR validation, and the expression of TRIM7, SGK196, BCL2A1, NR5A2, and ANK3 significantly differed between the two groups (Fig. 3c). These findings suggested for the first time that the expression of these genes may be directly mediated by MEF2A.

**Table 1:** GO annotation for the down-regulated DEGs with potential MEF2A binding sites in the proximal promoter

Category	Term	Count (%)	*p*-value	Fold enrichment
GAD_DISEASE_CLASS	Reproduction	8 (13.33)	0.03294	2.51
GAD_DISEASE_CLASS	Cardiovascular	21 (35)	0.03316	1.48
UP_KEYWORDS	Secreted	18 (30)	0.00002	3.14
UP_KEYWORDS	Disulfide bond	21 (35)	0.00097	2.10
UP_KEYWORDS	Signal	23 (38.33)	0.00181	1.90
UP_KEYWORDS	Glycoprotein	22 (36.67)	0.01248	1.66
UP_KEYWORDS	Cell junction	7 (11.67)	0.01257	3.56
UP_KEYWORDS	Cell membrane	16 (26.67)	0.03143	1.73
UP_KEYWORDS	Citrullination	3 (5)	0.03161	10.61
UP_KEYWORDS	Actin-binding	4 (6.67)	0.04397	5.01
UP_KEYWORDS	Thick filament	2 (3.33)	0.04765	40.35
UP_SEQ_FEATURE	Signal peptide	23 (38.33)	0.00012	2.30
UP_SEQ_FEATURE	Disulfide bond	19 (31.67)	0.00132	2.18
UP_SEQ_FEATURE	Zinc finger region:B box-type	3 (5)	0.01710	14.75
UP_SEQ_FEATURE	Glycosylation site:N-linked (GlcNAc.)	20 (33.33)	0.03095	1.58
UP_SEQ_FEATURE	Domain:B30.2/SPRY	3 (5)	0.03312	10.34

### DEGs in response to MEF2A knockdown were mostly enriched in the DAVID GO terms related to growth, signal transduction and inflammation

Excluding the DEGs that lacked the ENTREZ GENE ID information, we found that 384 and 578 of 962 DEGs were up-regulated and down-regulated, respectively (Additional file 2: Table S2). The annotation clusters containing GO terms, including glycoprotein, glycosylation site:N-linked (GlcNAc), signal peptide, disulfide bond, secreted, and signal, were significantly enriched in the DEGs (Additional file 3: Table S3). Functional GO term enrichment analysis revealed that preferential changes in the expression of the genes in response to MEF2A knockdown were involved in diverse biological processes, including “female pregnancy,” “cell–cell signaling,” and “metabolic process.” The DEGs were likely implicated in many molecular functions, including “phosphatidylinositol-4,5-bisphosphate 3-kinase activity,” “cytokine activity,” “serine-type endopeptidase inhibitor activity,” and “growth factor activity” (Table 2). The most significantly enriched GO terms in cellular component included extracellular space, extracellular region, and plasma membrane (Table 2). Pathway enrichment analysis showed that most of the DEGs down-regulated by the MEF2A knockdown were enriched in immune-related pathways (Table 3). These GO terms are closely associated with growth, development, cell survival, and inflammation, suggesting the extensive involvement of MEF2A in mediating cell functions.

**Table 2:** The top 10 enriched biological process, cell component and molecular function GO terms for DEGs, which were sorted respectively by *p*-value in ascending

Category	Term	Count	*p*-value	Fold enrichment
GOTERM_BP_DIRECT	GO:0007565 ~ female pregnancy	13	2.86E−04	3.534242
GOTERM_BP_DIRECT	GO:0007267 ~ cell–cell signaling	24	3.61E−04	2.286233
GOTERM_BP_DIRECT	GO:0008152 ~ metabolic process	18	5.93E−04	2.592425
GOTERM_BP_DIRECT	GO:0007165 ~ signal transduction	69	0.002168	1.438003
GOTERM_BP_DIRECT	GO:0001525 ~ angiogenesis	20	0.002302	2.170042
GOTERM_BP_DIRECT	GO:0008277 ~ regulation of G-protein coupled receptor protein signaling pathway	7	0.00493	4.342866
GOTERM_BP_DIRECT	GO:0030168 ~ platelet activation	12	0.007956	2.524796
GOTERM_BP_DIRECT	GO:0006687 ~ glycosphingolipid metabolic process	7	0.009997	3.763817
GOTERM_BP_DIRECT	GO:0071526 ~ semaphorin–plexin signaling pathway	6	0.010737	4.399266
GOTERM_BP_DIRECT	GO:0016266 ~ *O*-glycan processing	8	0.011451	3.226129
GOTERM_CC_DIRECT	GO:0005615 ~ extracellular space	109	1.54E−11	1.95324
GOTERM_CC_DIRECT	GO:0005576 ~ extracellular region	123	2.57E−11	1.844064
GOTERM_CC_DIRECT	GO:0005886 ~ plasma membrane	214	1.33E−04	1.253453
GOTERM_CC_DIRECT	GO:0005578 ~ proteinaceous extracellular matrix	25	3.33E−04	2.251656
GOTERM_CC_DIRECT	GO:0016021 ~ integral component of membrane	251	0.001739	1.17346
GOTERM_CC_DIRECT	GO:0005581 ~ collagen trimer	11	0.004686	2.886035
GOTERM_CC_DIRECT	GO:0009986 ~ cell surface	36	0.00631	1.603245
GOTERM_CC_DIRECT	GO:0005887 ~ integral component of plasma membrane	76	0.015603	1.296444
GOTERM_CC_DIRECT	GO:0002177 ~ manchette	3	0.015728	14.48265
GOTERM_CC_DIRECT	GO:0005884 ~ actin filament	8	0.017555	2.9708
GOTERM_MF_DIRECT	GO:0046934 ~ phosphatidylinositol-4,5-bisphosphate 3-kinase activity	10	8.46E−04	3.963234
GOTERM_MF_DIRECT	GO:0005125 ~ cytokine activity	18	8.47E−04	2.513051
GOTERM_MF_DIRECT	GO:0004867 ~ serine-type endopeptidase inhibitor activity	12	0.001915	3.039842
GOTERM_MF_DIRECT	GO:0008083 ~ growth factor activity	16	0.002531	2.426869
GOTERM_MF_DIRECT	GO:0008201 ~ heparin binding	15	0.005735	2.30363
GOTERM_MF_DIRECT	GO:0008401 ~ retinoic acid 4-hydroxylase activity	3	0.009368	18.42904
GOTERM_MF_DIRECT	GO:0005102 ~ receptor binding	25	0.009977	1.74023
GOTERM_MF_DIRECT	GO:0005509 ~ calcium ion binding	42	0.017368	1.439367
GOTERM_MF_DIRECT	GO:0032395 ~ MHC class II receptor activity	4	0.021097	6.552547
GOTERM_MF_DIRECT	GO:0001665 ~ alpha-*N*-acetylgalactosaminide alpha-2,6-sialyltransferase activity	3	0.022182	12.28603

**Table 3:** The top 10 significant KEGG pathway enriched in the down-regulated DEGs

Category	Term	Count	*p*-value	Fold enrichment
KEGG_PATHWAY	hsa05146:amoebiasis	11	9.00E−04	3.585377
KEGG_PATHWAY	hsa05164:influenza A	14	0.001468	2.779885
KEGG_PATHWAY	hsa04650:natural killer cell mediated cytotoxicity	11	0.002616	3.115164
KEGG_PATHWAY	hsa05323:rheumatoid arthritis	9	0.003677	3.533523
KEGG_PATHWAY	hsa05150:*Staphylococcus aureus* infection	7	0.004323	4.478704
KEGG_PATHWAY	hsa04612:antigen processing and presentation	8	0.006025	3.636842
KEGG_PATHWAY	hsa04610:complement and coagulation cascades	7	0.014078	3.505072
KEGG_PATHWAY	hsa05143:African trypanosomiasis	5	0.014207	5.234848
KEGG_PATHWAY	hsa05332:graft-versus-host disease	5	0.014207	5.234848
KEGG_PATHWAY	hsa04740:olfactory transduction	20	0.020697	1.73183

### PIK3CG and several chemotactic factors were included in the hot nodes in protein–protein interaction (PPI) networks

**Fig. 4:**
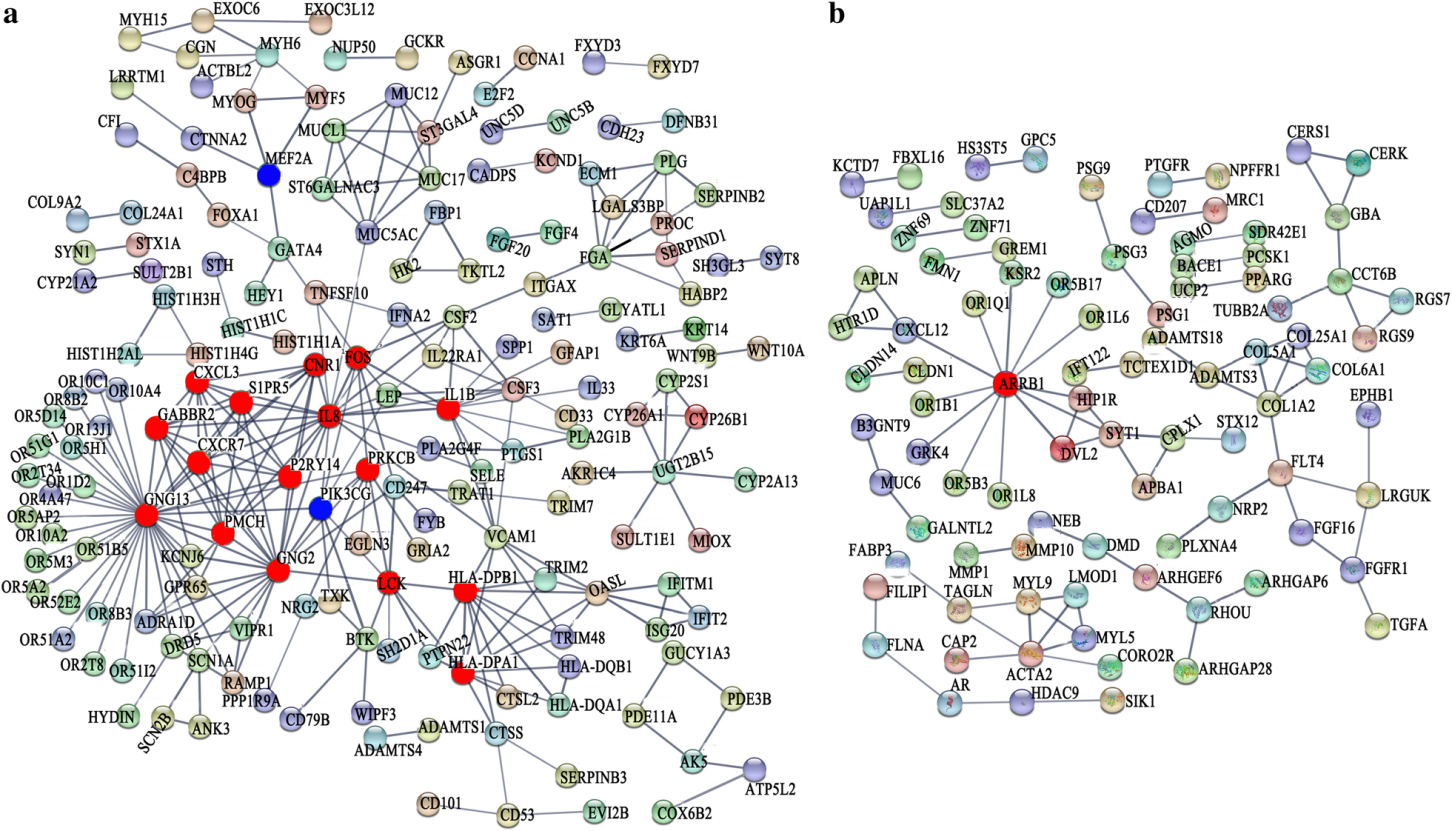
The functional association protein networks were constructed with STRING for the DEGs. Settings for the interaction search as follows: minimum required interaction score is ≥ 0.7 (high confidence); active interaction sources are Textmining, Experiments, Databases, Co-expression, Neighborhood, Gene Fusion and Co-occurrence; the max number of interactors to show is query proteins only for the first shell and none for the second shell; hide the disconnected nodes in the network. The pies indicated nodes and the full line indicated the edges of the PPI network. **a** Network for down-regulated genes. **b** Network for UP-regulated genes

The PPI network of the down-regulated DEGs contained 502 nodes and 281 edges, and the *p*-value of PPI enrichment was < 1.0 × 10^−16^. Most of the downregulated DEGs formed a complicated interaction network (Fig. 4a). GNG13, GNG2, GABBR2, CXCR7, CXCL3, IL8, CNR1, IL1B, PRKCB, PMCH, P2RY14, and PIK3CG were presented at the hot nodes and may play important roles in the biological process (Fig. 4a). The majority of the genes in the network were significantly enriched (FDR < 0.01) in the categories of extracellular space and extracellular region in the GO term of cellular component, and many of these genes were involved in signal transduction (Table 4). The KEGG pathways “amoebiasis, *Staphylococcus aureus* infections, influenza A, rheumatoid arthritis, and olfactory transduction” were significantly enriched (FDR < 0.05) for the DEGs in the PPI network (Table 4). However, most of the up-regulated genes are independent of each other and only a small number of them can form an interaction network (Fig. 4b).

**Table 4:** GO annotation for the down-regulated DEGs in the PPI network

Pathway ID	Pathway description	Count in gene set	False discovery rate (FDR)
Biological process (GO)			
GO:0050793	Regulation of developmental process	84	0.000902
GO:0048387	Negative regulation of retinoic acid receptor signaling pathway	8	0.00358
GO:0051239	Regulation of multicellular organismal process	88	0.00358
GO:0045595	Regulation of cell differentiation	60	0.00957
GO:0050995	Negative regulation of lipid catabolic process	6	0.00957
Cellular component (GO)			
GO:0005615	Extracellular space	66	8.23e−07
GO:0005576	Extracellular region	145	0.000952
GO:0071944	Cell periphery	143	0.0129
GO:0005886	Plasma membrane	139	0.0259
KEGG pathways			
05146	Amoebiasis	10	0.0173
05150	*Staphylococcus aureus* infection	7	0.0173
05164	Influenza A	14	0.0173
05323	Rheumatoid arthritis	9	0.0173
04740	Olfactory transduction	21	0.0413

**Fig. 5:**
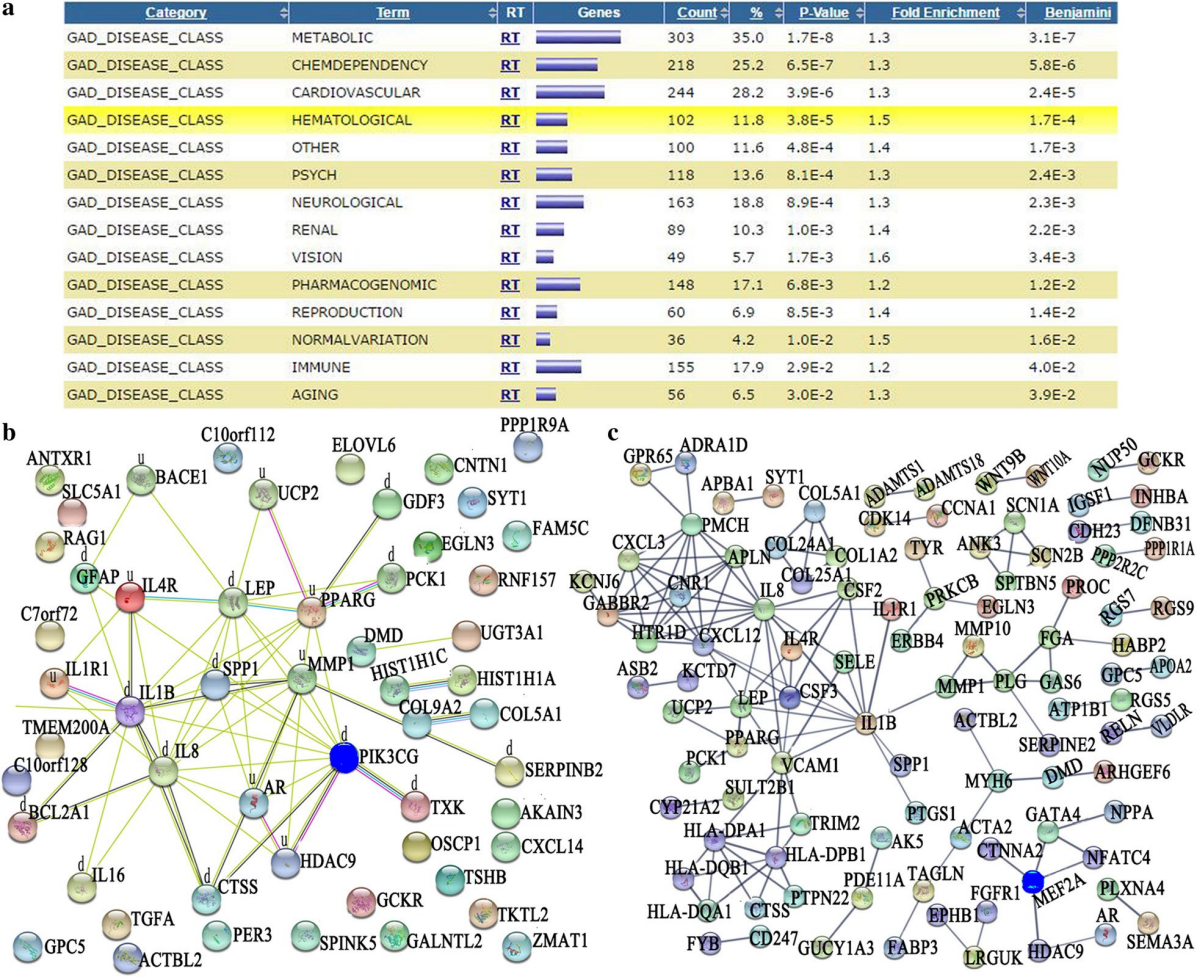
Enrichment of the GO terms in GAD_DISEASE_CLASS for the DEGs and PPI network for certain GO term ‘aging’ and ‘cardiovascular’ related DEGs. In the PPI network, each node represents all the proteins produced by a single, protein-coding gene locus. Edges represent protein–protein associations, and associations are meant to be specific and meaningful. **a** The Go terms significantly enriched for the DEGs. **b** Interaction network for the DEGs enriched in the GO term ‘aging’. A “d” indicates a gene with down-regulatory signal on the microarray. A “u” indicates a gene with up-regulatory signal on the microarray. **c** Interaction network for the DEGs enriched in the GO term ‘cardiovascular’

**Fig. 6:**
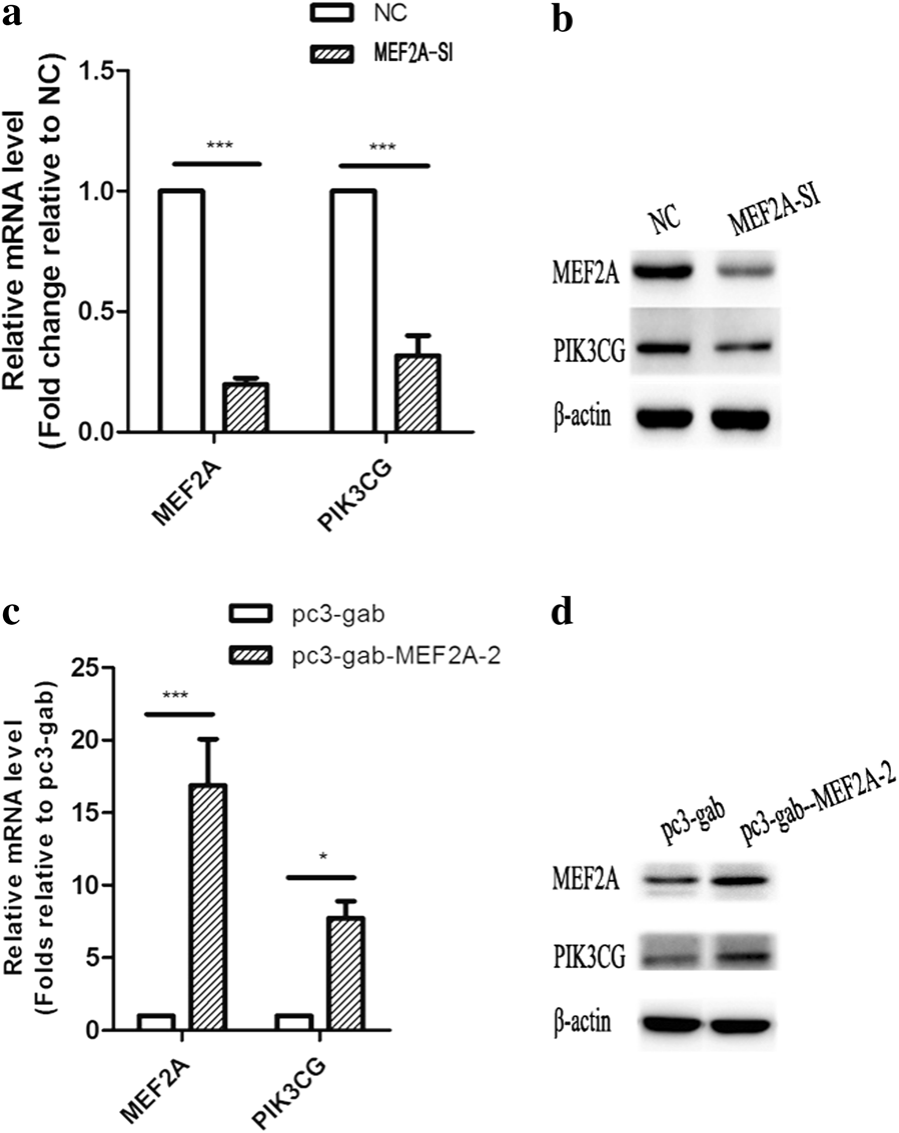
The expression of PIK3CG varies with the level of MEF2A. **a**, **b** Changes in the expression of PIK3CG at the mRNA and protein levels when MEF2A is inhibited; NC: transfected with negative control siRNA; MEF2A-SI: transfected with MEF2A specific siRNA (MEF2A-1527). **c**, **d** Changes in the expression of PIK3CG at mRNA and protein levels when MEF2A was over-expressed

In order to discover the genes possibly involved in related diseases, the DEGs were annotated to the category “GAD_DISEASE_CLASS” and the terms “metabolic, chemdependency, cardiovascular, hematological, psych, neurological, renal, vision, pharmacogenomic, reproduction, normal variation, immune, aging, and developmental” were preferentially enriched (Fig. 5a). Among the DEGs in the GO term “aging,” 23 were up-regulated and 33 were down-regulated. The PPI network of the DEGs in the GO term “aging” showed that 22 DEGs, including PIK3CG, TXK, HDAC9, PPARG, IL1B, IL8, and PCK1, were in a correlative interaction network (Fig. 5b). A PPI network analysis performed for 244 DEGs in the GO term “cardiovascular” showed that these genes had more interactions among themselves than the expected observation (PPI enrichment *p* < 1 × 10^−16^), suggesting that the genes were biologically connected as a group (Fig. 5c). PIK3CG, IL8, IL1B, and CSF were included in the hot nodes with multiple interactions, implying their major role in the GO terms “aging” and “cardiovascular.” Further validation experiments showed that the mRNA and protein levels of PIK3CG were significantly down-regulated when MEF2A was inhibited (Fig. 6a), while the mRNA and protein levels of PIK3CG were significantly up-regulated when overexpressing MEF2A (Fig. 6b).

## Discussion

In the present study, inhibition of MEF2A by siRNA promoted HCAEC senescence, suggesting that it has an anti-aging functional role. This protective role appears to be different from its role in VSMC, which exhibits a detrimental aging-promoting role [16, 17]. However, an in vivo research has shown that MEF2A may have an anti-atherosclerotic function in ApoE^−/−^ mice [19], and functional deletion in MEF2A gene is co-segregated with premature CAD in several family lineages [7, 8], suggesting the protective role of MEF2A in vessels. The above findings imply that a decrease in MEF2A expression in vascular endothelial cells may result in vessel dysfunction. Therefore, it is important to understand the profile and the interaction network of the DEGs affected by knockdown of MEF2A in HCAEC.

In our study, the DEGs in response to MEF2A knockdown were significantly enriched in the GO terms associated with growth, proliferation, development, survival, and inflammation. The down-regulated genes in MEF2A-knocked down HCAECs were involved in a complicated regulatory network. Among the hot nodes, PIK3CG, PRKCB, and IL8 have been extensively investigated. PIK3CG plays important roles in immunity, hypertension, longevity, and circulation [21, 22]. p110γ, the encoding protein of PIK3CG, is principally involved in the control of leukocyte chemotaxis and inflammatory reactions in the immune system, affects multiple aspects of adrenergic receptor signaling and cardiac function in cardiomyocytes [21], and participates in angiotensin II induced calcium influx and governing the vascular contractile response [22]. Thus, p110γ has been considered as a promising target for drug design in the treatment of heart failure and hypertension, whereas the function of p110γ in vascular endothelial cells is unclear. In our study, the GO term analysis based on the GAD_disease indicated that PIK3CG participated in the aging pathway and interacted with many inflammatory factors in the PPI network. In practice, mass evidences have indicated that PI3K pathway is involved in cellular senescence and proliferation [23–26]. In the present study, multiple experiments showed that the expression of PIK3CG varied with the expression level of MEF2A, suggesting that the expression of PIK3CG is regulated by MEF2A. The expression of PIK3CG was significantly down-regulated in MEF2A-knocked down HCAECs, suggesting that PIK3CG may be involved in the molecular mechanism of MEF2A silencing induced senescence. In addition, many inflammatory factors were overtly down-regulated simultaneously with PIK3CG, indicating that the down-regulation of inflammatory factors in response to MEF2A silencing might be mediated by PIK3CG.

PRKCB might promote the removal of LDLs from the blood by increasing the expression of LDLR and SR-A and reducing foam cell formation, which might mitigate atherogenesis. However, the characteristics of PRKCB that plays a pro-inflammatory role in endothelial cells and promotes VSMC proliferation and migration are associated with pro-atherosclerosis as observed in PRKCB knockout mice [27].

IL8 is another extensively studied gene that serves as a risk factor of cardiovascular disease [28]. The PPI network shows a large number of interactions between IL8 and other genes, suggesting that MEf2A-mediated inflammatory pathway might greatly depend on the participation of IL-8. Suzuki et al. [29] revealed that MEF2A may mediate vascular inflammation by stimulating MCP-1 expression in VSMCs and macrophage infiltration. MEF2A and MEF2D may function as activators or repressors of gene transcription in human macrophages [30]. In our study, many genes involved in macrophage infiltration were downregulated in MEF2A-knocked down HCAECs, suggesting the role of MEF2A in regulating inflammation. Furthermore, 8 of the top 10 KEGG pathways enriched by the down-regulated DEGs were involved in immune-related diseases, indicating that MEF2A participated in mediating the immune system.

Guanine nucleotide binding protein subunit gamma 2 and 13 (GNG2 and GNG13) play key roles in signal transduction pathways. In our study, they were significantly down-regulated in MEF2A-knocked down HCAECs. GNG13 is colocalized with alpha-gustducin in taste receptor cells, and antibodies against Ggamma13 block the denatonium-induced increase in inositol trisphosphate (IP3) in taste tissue [31]. In our study, most olfactory receptors were distinctively down-regulated in MEF2A-knocked down HCAECs, and almost all of them interacted with GNG13 in the PPI network. These findings suggested that the expression of olfactory receptors might be regulated by MEF2A through GNG13 and PI3K.

DAVID GO functional annotation enrichment based on GAD revealed that the cardiovascular system was one of the most prominently enriched terms of the DEGs in response to MEF2A knockdown. More than 200 DEGs were related to the cardiovascular system and exhibited complicated interactions with one another. Many hot nodes in the PPI network are inflammatory factors, including IL8, IL1B, CXCL3, CXCL12, and HLA genes, which are associated with cardiovascular diseases [28, 32–34]. MEF2A functional deletion carriers were subjected to premature CAD [7, 8], strongly supporting the important roles of MEF2A in providing protection against cardiovascular disease. Our findings show that numerous genes in inflammatory pathways were down-regulated as MEF2A was silenced, indicating that MEF2A may be an important regulator to mediate the expression of inflammatory factors, and that this role of MEF2A appears to be inconsistent with its protective effect against CAD. Alternative splicing of the pre-mRNA of MEF2A resulted in different isoforms which have distinct activity and are differentially distributed in various tissues [35]. Aberrant alternative splicing isoforms were observed to be associated with myotonic dystrophy and neuromuscular disorders [36]. More than 20 MEF2A isoforms have been submitted to NCBI database so far. Yet the functional diversity of the isoforms is unclear. Silencing of MEF2A by siRNA may alter the proportion of different splicing isoforms compared to in the normal physiological status of the cells. Therefore, when we attempt to explore the underlying molecular mechanism that MEF2A participate in a certain biological or pathological process, which MEF2A alternative splicing isoforms exert major effect on the process should be taken into account. We found that the functional role of MEF2A in vascular endothelial cells is inconsistent with its roles in VSMC reported by other studies, possibly due to differential splicing of MEF2A in the two kinds of cells.

Among the down-regulated DEGs, 61 genes were found with at least one potential MEF2A binding site in the proximal promoter. If extended the search region in the promoters of the DEGs, there would be more genes found with MEF2A binding sites. Therefore, many DEGs may be directly regulated by MEF2A via binding to their cis-elements in the promoters. TRIM7, SGK196, BCL2A1, NR5A2, ANK3 and PYHIM9 have MEF2A binding site in their proximal promoter, which were down-regulated in MEF2A-knocked down HCAEC via verifying by qPCR. These genes are respectively involved in metabolism, development, differentiation, apoptosis and survival [37, 38].

## Conclusion

MEF2A knockdown in HCAECs promotes cell senescence and remarkably alters mRNA profile, primarily related to signal transduction, proliferation, development, inflammation, and other processes, and may lead to cell dysfunction by promoting cellular senescence and inflammation or by inhibiting cell proliferation. PIK3CG has been shown to be associated with longevity, and its expression varies with the expression level of MEF2A in HCAEC, suggesting that PIK3CG may play an important role in cell senescence induced by MEF2A silencing.

## Materials and methods

### Cell culture

The primary HCAECs were purchased from Cell Applications and cultured in EGM-2 medium (Lonza, USA) supplemented with growth factors. The cells were further grown at 37 °C in a humidified atmosphere with 5% (v/v) CO_2_ and passaged at 1:3 when they reached approximately 85–95% confluency.

### Construction of MEF2A overexpression plasmid

The complete MEF2A coding region fragment was amplified using high fidelity PCR polymerase and cDNA template derived from HCAEC, and the amplified fragment was ligated into the mammalian expression vector pc3-gab at the sites *Eco*RI and *Bgl*II. Then, transformation and plasmid extraction were performed, and the inserted encoding sequence was verified by Sanger sequencing to be same to the MEF2A alternative splice variant XM_005254915.2_V_X15.

### Transfection

siRNA was diluted to a 20 μM solution with RNAse-free and sterile deionized water and stored below − 20 °C. HCAECs were seeded in a plate at a density of 7500 cells/cm^2^ 18–24 h before transfection and cultured at 37 °C in a humidified atmosphere with 5% (v/v) CO_2_. Lipofectamine^®^ RNAiMAX and Lipofectamine^®^ 3000 reagent were respectively used to guide siRNA and plasmids into the cells, and the procedure was performed in accordance with the manufacturer’s instructions. The next treatments were performed at 72 h post-transfection.

### Detection of cell senescence and proliferation

The cells were subjected to SA-β-Gal staining by using a β-galactosidase staining kit (Cell Signaling Technology, USA) in accordance with the manufacturer’s instructions. The stained cells were examined with a high-resolution microscope at 400× magnification, and blue stain corresponded to senescent cells. Cell proliferation was detected with an EdU staining kit (Ribobio, China) in accordance with the manufacturer’s instructions. The stained cells were imaged with a fluorescence inversion microscope system at 400× magnification, and purple stain indicated proliferating cells. The senescence rate and the proliferation rate were respectively determined by calculating the proportion of the senescent cells and the proliferating cells in the total number of cells in 15 visions per well. The results were expressed as the mean of triplicates ± standard error of the mean (SEM).

### RNA isolation

Total RNA of cells was isolated with Trizol reagent (Invitrogen, Carlsbad, USA) in accordance with the manufacturer’s protocol. RNA concentration was determined by measuring the absorbance at 260 nm with a NanoVue spectrophotometer (GE Healthcare, UK). RNA integrity was assessed through electrophoresis on agarose gel.

### Microarray analysis

Three independent groups of HCAECs transfected with MEF2A-specific siRNA (MEF2A-Homo-1527) were separately analyzed with an Agilent human mRNA array (AHMA), and three independent groups of HCAECs transfected with negative control siRNA were collected together into a tube. RNA was extracted for AHMA analysis. Three MEF2A-specific siRNA groups were indicated as 1527-1, 1527-2, and 1527-3, and the mixed control group was designated as NC. The AHMA was designed with eight identical arrays/slide (8 × 60 K format), and each array contained probes examining about 27,958 Entrez gene RNAs. The whole microarray analyses, including sample preparation, labeling, hybridization, scanning, and treatment of original data, were processed by CapitalBio Corporation (Beijing, China). DEGs were analyzed by using the DAVID v6.8, and a protein–protein interaction (PPI) network was searched using the STRING tool [39]. The DEGs containing a potential MEF2A binding site in their promoter were predicted with LASAGNA-Search 2.0 [40].

### Real-time fluorescence quantitative PCR (q-PCR)

Total RNA was reverse transcribed by using a ReverTra Ace qPCR RT kit (Toyobo, Japan), and q-PCR was performed with SYBR^®^ Green mix (Takara, China) in accordance with the manufacturer’s instructions. Alteration in the gene expression relative to the control group was analyzed with a comparative CT method by using beta-actin for endogenous control and NC cell for calibration. The primers used in this study are shown in Table 5.

**Table 5:** The primer sequences for real time quantitative PCR

Serial no.	Genes	Forward primer (5′ to 3′)	Reverse primer (5′ to 3′)	Tm (°C)
1	MEF2A	AGCAGCCCTCAGCTCTCTTG	GGTGAAATCGGTTCGGACTTG	60
2	PIK3CG	TCCTCTTTGTGATGGGAACTT	TGTGTGATGACGAAGGGCT	60
3	SERPIND1	TGATTCTCAACTGCATCTACTTC	CCTCTCTCTCATTCAGCCG	60
4	IL1B	ACAGTGGCAATGAGGATG	TGTAGTGGTGGTCGGAGA	60
5	ENPP1	ACTGCGAAAGTATGCTGA	TTCTTGGTTACGGATGAC	60
6	ZAK	GCGGTGGAGGAAGATTTG	TGACTGAGGACCCTGAGTATTT	60
7	FOS	TACTACCACTCACCCGAAGA	GAATGAAGTTGGCACTGGA	58
8	MMP1	AAACCCCAAAAGCGTGTG	GGGTAGAAGGGATTTGTGC	60
9	DQB1	CTGGTGATGATGGAAATGAC	TTGCTCTGGGCAGATTCA	60
10	GUCY1A3	GGAGCCGAGTCTATCTTCA	TGTGCTGTGCCAATGTTC	60
11	MYL9	AACGCCTTTGCCTGCTTC	CATCTCGTCCACTTCCTCATC	60
12	GJA5	GGCAAGGAAATAGTAGGG	TTGGCAGTCTCATTAGGG	60
13	CSF2	CCTGTGCAACCCAGATTA	TGGCTACCAGCAGTCAAA	60
14	TRIM	GGGTGTCCACATAATTGTTGT	GCCCTAACTGGTATGTTACG	60
15	MRPL45	GAAATGTTGTCAGGGTGTTG	CCTTAAAGTCACATCGCTCA	60
16	SGK196	ACCTGGAAGAAACACTAAACC	GGTCGTTGGAGTCGCACA	60
17	BCL2A1	CATCATTAACTGGGGAAGAA	ATTATGAACTCCGCAACAAA	60
18	NR5A2	CGTGGAGGAAGGAATAAG	TCAGGTCAGAGGGCATAG	60
19	ANK3	CCTATTTATGTTGATTGTTATGG	CTTGGCTGGTGTCTCTAATCT	60
20	PYHIN1	AAGGAGAATGCCACAATATC	TGTCTCTGGATGAAACTATGC	60

### Statistical analysis

Data were expressed as mean ± SEM with more than three independent experiments. The statistical significance of the comparison between groups was examined with Student’s t test, and *p* < 0.05 indicates significant differences.

## Additional files

**Additional file 1: Table S1.** The information of the predicted MEF2A binding sites on the proximal promoter of the DEGs.

**Additional file 2: Table S2.** The genes with more than twofold change in expression in the siRNA group compared to the negative control. Up, up-regulated in MEF2A specific siRNA group; down, down-regulated in MEF2A specific siRNA group.

**Additional file 3: Table S3.** Annotation for the differentially expressed genes.

## Abbreviations

MEF2A: myocyte enhancer factor 2A
HCAEC: human coronary artery endothelial cells
DEG: differentially expressed genes
GO: gene ontology
KEGG: Kyoto Encyclopedia of Genes and Genomes
CAD: coronary artery disease
SA-β-Gal: senescence-associated β-galactosidase
VSMC: vascular smooth muscle cell
PAH: pulmonary arterial hypertension
LDL: low density lipoprotein cholesterol
LDLR: low density lipoprotein cholesterol receptor
PPI: protein–protein interaction
siRNA: small interference RNA
AHMA: Agilent human mRNA array
DAVID: Database for Annotation, Visualization, and Integrated Discovery
